# Supplementary material for: High Mortality in an Older Japanese Population with Low Forced Vital Capacity and Gender-Dependent Potential Impact of Muscle Strength: Longitudinal Cohort Study
Source: J Clin Med. 2022 Sep 6;11(18):5264. doi: 10.3390/jcm11185264 (PMC9505108; doi:10.3390/jcm11185264)
Supplement: Supplementary file 1 [file jcm-11-05264-s001.zip › jcm-1849961-supplementary.pdf]

Supplementary Table S1: Association between FVC% predicted and mortality (Exclusion of deaths occurring in the first 3 years of follow-up) *.

	**Males (410)**				
	Q1[**]	Q2[††]	Q3[‡‡]	Q4[§§]	*P*-trend
**Number of cases**	103	103	105	99	
**Number of death**	29	35	34	46	
**Model 1[†]**	1.0 (ref.)	1.33 (0.81–2.18)	1.19 (0.72–1.95)	1.99 (1.25–3.16)	0.010
**Model 2[‡]**	1.0 (ref.)	1.33 (0.81–2.17)	1.19 (0.72–1.95)	1.94 (1.22–3.10)	0.012
**Model 3[§]**	1.0 (ref.)	1.55 (0.93–2.60)	1.25 (0.74–2.14)	2.10 (1.27–3.47)	0.014
	**Females (560)**				
	Q1 [**]	Q2[††]	Q3[‡‡]	Q4[§§]	*P*-trend
**Number of cases**	141	141	139	139	
**Number of death**	22	31	20	30	
**Model 1[†]**	1.0 (ref.)	1.55 (0.90–2.67)	0.95 (0.52–1.75)	1.42 (0.82–2.47)	0.520
**Model 2[‡]**	1.0 (ref.)	1.71 (0.99–2.97)	1.08 (0.59–1.99)	1.48 (0.86–2.58)	0.418
**Model 3[§]**	1.0 (ref.)	1.75 (0.98–3.11)	1.04 (0.54–1.99)	1.48 (0.82–2.70)	0.494

FVC% predicted = % predicted value forced vital capacity; BMI = body mass index; hs-CRP = high sensitive C-reactive protein; ref = reference. * Hazard ratio (95% confidence interval). [†] Model 1: univariate model.[‡] Model 2: adjusted for age. [§] Model 3: adjusted for Model2 + medical history (pneumonia, bronchial asthma, cancer, stroke, myocardial infarction, dia-betes, hypertension), smoking, alcohol consumption, depressive symptoms, cognitive function, educational level, marital status, leg extension power, BMI, albumin and hs-CRP. [**] Q1; FVC% predicted ≥100.5 (male), ≥114.5 (female). [††]Q2; FVC% predicted ≥89.2 to <100.5 (male), ≥100.9 to <114.5 (female). [‡‡]Q3; FVC% predicted ≥78.3 to <89.2 (male), ≥87.7 to <100.9 (female). [§§]Q4; FVC% predicted <78.3 (male), <87.7 (female).

Supplementary Table S2: Association between FVC% predicted (2 groups <80% and ≥80%) and mortality *.

	**Males (420)**			**Females (565)**		
	%FVC ≥80%	%FVC <80%	*P*	%FVC ≥80%	%FVC <80%	*P*
**Number of cases**	296	124		484	81	
**Number of death**	97	57		85	23	
**Model 1[†]**	1.0 (ref.)	1.60 (1.15–2.22)	0.005	1.0 (ref.)	1.67 (1.05–2.64)	0.030
**Model 2[‡]**	1.0 (ref.)	1.57 (1.13–2.18)	0.007	1.0 (ref.)	1.63 (1.03–2.58)	0.038
**Model 3[§]**	1.0 (ref.)	1.58 (1.11–2.24)	0.010	1.0 (ref.)	1.87 (1.11–3.14)	0.019

FVC% predicted = % predicted value forced vital capacity; BMI = body mass index; hs-CRP = high sensitive C-reactive protein, ref = reference. * Hazard ratio (95% confidence interval). [†] Model 1: univariate model. [‡] Model 2: adjusted for age. [§] Model 3: Model 2 adjusted for medical history (pneumonia, bronchial asthma, cancer, stroke, myocardial infarction, dia-betes, hypertension), smoking, alcohol consumption, depressive symptoms, cognitive function, educational level, marital status, leg extension power, BMI, albumin and hs-CRP.

Supplementary Table S3: The effect of muscle strength (2 groups) * on the association between FVC% predicted (2 groups <80% and ≥80%) and mortality [†].

	**Males (420)**			**Females (565)**		
	FVC% predicted ≥80%	FVC% predicted <80%	*P*	FVC% predicted ≥80%	FVC% predicted <80%	*P*
**Model 1 [‡]**						
FVC% predicted	1.0 (ref.)	1.60 (1.15–2.22)	0.005	1.0 (ref.)	1.67 (1.05–2.64)	0.030
Leg power		-	-		-	-
**Model 2 [§]**						
FVC% predicted	1.0 (ref.)	1.48 (1.06–2.06)	0.021	1.0 (ref.)	1.67 (1.05–2.67)	0.030
Leg power		1.67 (1.09–2.54)	0.018		0.98 (0.63–1.52)	0.912
**Model 3 [‖]**						
FVC% predicted	1.0 (ref.)	1.59 (1.12–2.25)	0.009	1.0 (ref.)	1.81 (1.08–3.02)	0.025
Leg power		1.70 (1.07–2.68)	0.023		1.05 (0.61–1.79)	0.861

FVC% predicted = %forced vital capacity; BMI = body mass index; hs-CRP = high sensitive C-reactive protein; ref = reference. * Leg extension power ≥15.8 w/kg (male), ≥9.9 w/kg (female) and leg extension power <15.8 w/kg (male), <9.9 w/kg (female). [†] Hazard ratio (95% confidence interval). [‡] Model 1: univariate model. [§] Model 2: Model 1+ leg extension power. [‖] Model3: Model 2 adjusted for age, medical history (pneumonia, bronchial asthma, cancer, stroke, myocardial infarction, diabetes, hypertension), smoking, alcohol consumption, depressive symptoms, cognitive function, educational level, marital status, BMI, albumin and hs-CRP.
